# Pleiotrophin Activates cMet- and mTORC1-Dependent Protein Synthesis through PTPRZ1—The Role of α_ν_β_3_ Integrin

**DOI:** 10.3390/ijms251910839

**Published:** 2024-10-09

**Authors:** Eleni Mourkogianni, Katerina Karavasili, Athanasios Xanthopoulos, Michaela-Karina Enake, Lydia Menounou, Evangelia Papadimitriou

**Affiliations:** Laboratory of Molecular Pharmacology, Department of Pharmacy, University of Patras, 26504 Patras, Greece; eleni9119@yahoo.gr (E.M.); up1079146@upatras.gr (K.K.); athanasiosxan@gmail.com (A.X.); mihaelaenake92@gmail.com (M.-K.E.); up1073880@upnet.gr (L.M.)

**Keywords:** angiogenesis, endothelial cells, integrin, mTOR, pleiotrophin, tyrosine kinase, tyrosine phosphatase

## Abstract

Pleiotrophin (PTN) is a secreted factor that regulates endothelial cell migration through protein tyrosine phosphatase receptor zeta 1 (PTPRZ1) and α_v_β_3_ integrin. Genetic deletion of *Ptprz1* results in enhanced endothelial cell proliferation and migration, due to the decreased expression of β_3_ integrin and the subsequent, enhanced cMet phosphorylation. In the present study, we investigated the effect of PTN and PTPRZ1 on activating the mTORC1 kinase and protein synthesis and identified part of the implicated signaling pathway in endothelial cells. PTN or genetic deletion of *Ptprz1* activates protein synthesis in a mTORC1-dependent manner, as shown by the enhanced phosphorylation of the mTORC1-downstream targets ribosomal protein S6 kinase 1 (SK61) and 4E-binding protein 1 (4EBP1) and the upregulation of HIF-1α. The cMet tyrosine kinase inhibitor crizotinib abolishes the stimulatory effects of PTN or PTPRZ1 deletion on mTORC1 activation and protein synthesis, suggesting that mTORC1 activation is downstream of cMet. The mTORC1 inhibitor rapamycin abolishes the stimulatory effect of PTN or PTPRZ1 deletion on endothelial cell migration, suggesting that mTORC1 is involved in the PTN/PTPRZ1-dependent cell migration. The α_v_β_3_ integrin blocking antibody LM609 and the peptide PTN_112–136_, both known to bind to α_ν_β_3_ and inhibit PTN-induced endothelial cell migration, increase cMet phosphorylation and activate mTORC1, suggesting that cMet and mTORC1 activation are required but are not sufficient to stimulate cell migration. Overall, our data highlight novel aspects of the signaling pathway downstream of the PTN/PTPRZ1 axis that regulates endothelial cell functions.

## 1. Introduction

Pleiotrophin (PTN) is an 18 kDa heparin-binding secreted factor that was initially shown to be a pro-angiogenic molecule but was later proved to also act as an endogenous brake for the excess angiogenesis induced by vascular endothelial growth factor A (VEGFA) [1,2]. Although PTN has been shown to bind to numerous cell surface receptors [1], it seems that the protein tyrosine phosphatase receptor zeta 1 (PTPRZ1) plays a central role in its stimulatory effects on endothelial cells [3]. In contrast, the binding of PTN to the VEGF receptor 2 (VEGFR2) competes with VEGFA binding and limits the angiogenic effects of VEGFA [4]. The pivotal role of PTPRZ1 in PTN signaling was recently shown using mice that were knockout for PTPRZ1. Genetic deletion of *Ptprz1* enhances angiogenesis in vivo and in vitro [3,5]. PTN and a selective PTPRZ1 tyrosine phosphatase inhibitor stimulate migration in *Ptprz1*^+/+^ endothelial cells, while in *Ptprz1^−/−^* endothelial cells, they have no further stimulatory effects, supporting the notion that PTN acts by inhibiting the PTPRZ1 tyrosine phosphatase activity. Downstream of PTPRZ1, PTN activates the tyrosine kinase receptor cMet, which seems to be a key mediator of the PTN/PTPRZ1 effects on endothelial cell activation and angiogenesis [3].

mTOR is a serine/threonine kinase crucial for cell adaptation to various environmental stimuli. It acts as a sensor for nutrients, growth factors, and cellular energy availability, and regulates transcription, translation, senescence, and survival, via the phosphorylation of numerous substrates. mTOR is a component of two distinct multiprotein complexes, mTOR complex 1 (mTORC1) and mTORC2, composed of different protein partners that are differentially activated and have distinct roles in cellular signaling [6,7,8]. The main function of mTORC1 is to regulate protein synthesis and cell growth through the phosphorylation of the downstream molecules eukaryotic translation initiation factor 4E binding protein 1 (4EBP1) and ribosomal protein S6 kinase 1 (S6K1) that have been generally accepted as markers of activated mTORC1 signaling [9,10]. mTORC1 regulates anabolic processes, including ribosome biogenesis and protein synthesis [11]. The macrolide rapamycin is an inhibitor of mTORC1, and rapamycin or its analogs (rapalogs) inhibit T-cell activation and are qualified to prevent organ transplant rejection. Rapalogs also inhibit tumor cell growth and are being used or studied for treating different types of tumors with hyperactive mTORC1 signaling [12,13]. Although rapamycin is selective for mTORC1 compared with mTORC2 due to structural differences between the two complexes, it can also inhibit mTORC2 after prolonged treatment in vitro [14,15,16], and in vivo [16,17].

Recent studies have shown that PTN, and genetic deletion, or tyrosine phosphatase inhibition of PTPRZ1 activate the PI3K/AKT/mTOR pathway in oligodendrocytes, contributing to the accelerated differentiation of oligodendrocytes and the earlier remyelination after cuprizone-induced demyelination [18]. Similarly, PTN rescued brain white matter injury in neonatal rats by activating mTOR and enhancing oligodendrocyte differentiation and myelination [19]. Microglia-associated PTN may also mediate the regulation of oligodendrocyte precursor cells by activating the mTOR signaling pathway in a mouse subarachnoid hemorrhage model [20]. In the present study, we investigated whether PTN activates mTORC1 and protein synthesis in endothelial cells and elucidated part of the implicated signaling pathway, focusing on its impact on endothelial cell migration and the potential of its pharmacological targeting.

## 2. Results

### 2.1. PTN Activates mTORC1 and Protein Synthesis in Endothelial Cells

**Figure 1:**
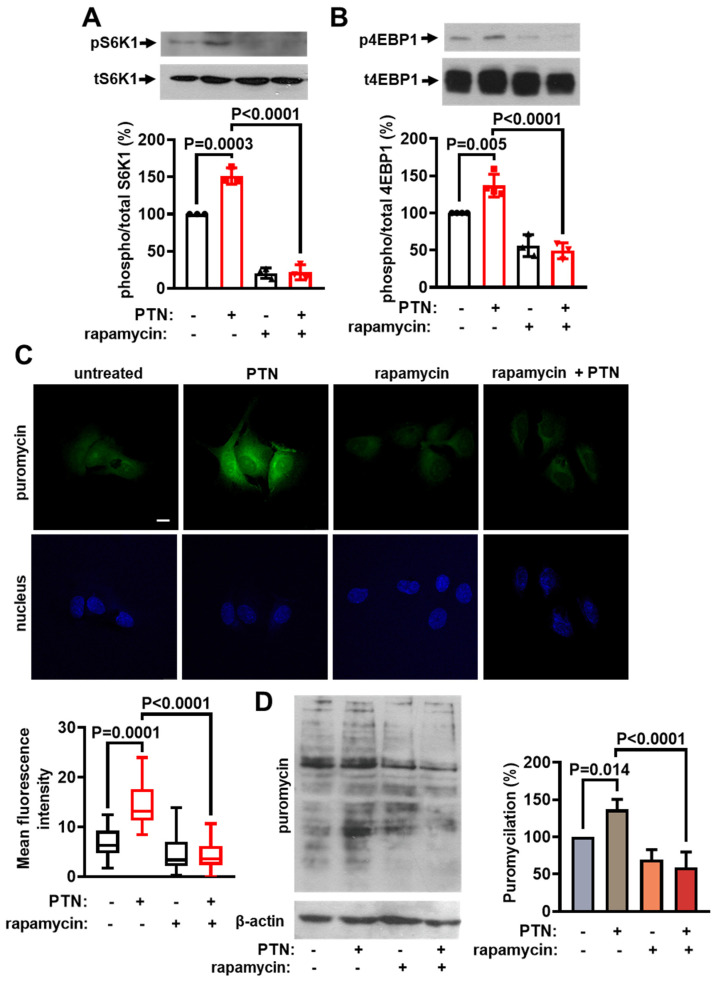
PTN activates mTORC1 and protein synthesis in endothelial cells. Serum-starved HUVEC were stimulated with 100 ng/mL PTN for 10 min in the presence or absence of 20 nM rapamycin. Rapamycin was added to the cells 15 min before PTN stimulation. (**A**,**B**) Representative Western blot images of phosphorylated and total S6K1 and 4EBP1 in HUVEC. The bands were quantified, and the results are expressed as the mean ± standard deviation of the % ratio of phosphorylated to total protein compared to the corresponding control. The bullets on the graphs indicate independent assays. Control corresponds to cells incubated with 0.0002% DMSO (rapamycin solvent) and is considered 100%. (**C**) Representative confocal microscopy images of serum-starved HUVEC treated with PTN for 2 h. A cell-permeable puromycin analog was added for 30 min to label the newly synthesized peptides. The puromycin analog incorporated into newly synthesized proteins of HUVEC is shown in green, and the nuclei stained with Draq5 are shown in blue. The scale bar corresponds to 10 μm. Box plots indicate the median, mean, and range of the detected green fluorescence signal per cell (8–10 image fields with 4–8 cells per image, per sample, n = 3). (**D**) Representative Western blot images of the newly synthesized peptides labeled with puromycin from total cell lysates from serum-starved HUVEC treated with PTN for 2 h in the presence or absence of rapamycin. Puromycin (1 μΜ) was added for the last 10 min. Beta (β)-actin was used as a loading control. Results are expressed as the mean ± standard deviation (n = 4) of the % ratio of puromycin incorporation (puromycilation) compared to the control (cells incubated with 0.0002% DMSO, considered 100%).

To investigate whether PTN activates mTORC1 in endothelial cells, we studied the phosphorylation levels of the mTORC1 substrates S6K1 and 4EBP1 in human umbilical vein endothelial cells (HUVEC). PTN enhanced the phosphorylation of both S6K1 (Figure 1A) and 4EBP1 (Figure 1B), suggesting mTORC1 activation. PTN increased protein synthesis in endothelial cells, as measured by a puromycin incorporation assay in HUVEC (Figure 1C,D) and mouse lung microvascular endothelial cells (LMVEC, Figure S1). The mTORC1 pharmacological inhibitor, rapamycin, abolished PTN-induced phosphorylation of S6K1 and 4EBP1 and protein synthesis (Figure 1), further supporting the mTORC1 activation by PTN and its role in translation initiation.

### 2.2. Ptprz1 Genetic Deletion Activates mTORC1 and Protein Synthesis in Endothelial Cells

**Figure 2:**
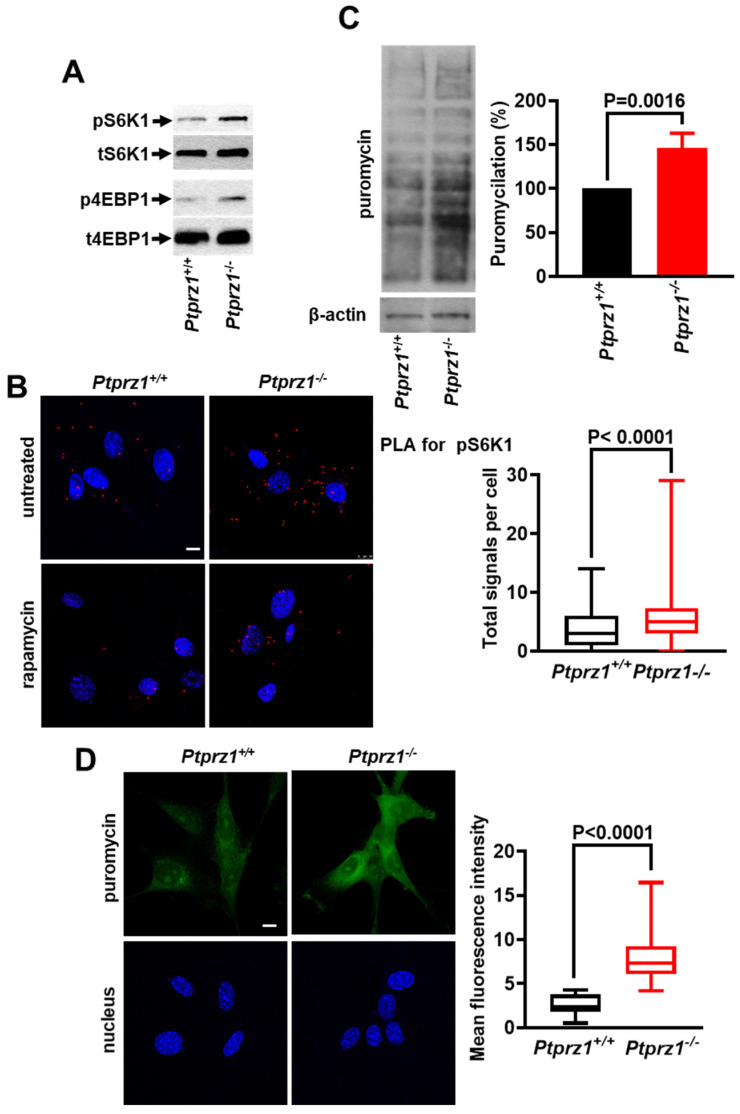
*Ptprz1* genetic deletion activates mTORC1 and protein synthesis in endothelial cells. (**A**) Representative Western blot images of phosphorylated and total S6K1 and 4EBP1 in *Ptprz1^+/+^* and *Ptprz1^−/−^* LMVEC cultured in full medium. (**B**) Representative confocal microscopy images and quantification of a PLA assay for detecting pS6K1 (red dots) in *Ptprz1^+/+^* and *Ptprz1^−/−^* LMVEC cultured in full medium. Nuclei stained with Draq5 are shown in blue. The scale bar corresponds to 10 μm. Box plots indicate the median, mean, and range of detected signals (8–10 image fields with 4–8 cells per image, per sample, n = 3). (**C**) Representative Western blot images of the newly synthesized peptides labeled with puromycin in total cell lysates from LMVEC cultured in a full medium, 10 min following the addition of puromycin. Beta (β)-actin was used as a loading control. Results are expressed as the mean ± standard deviation (n = 4) of the % ratio of puromycin incorporation (puromycilation) compared to the *Ptprz1^+/+^* LMVEC (considered 100%). (**D**) Representative confocal microscopy images and quantification of the cell-permeable puromycin analog incorporated into newly synthesized proteins of *Ptprz1^+/+^* and *^−/−^* LMVEC cultured in full medium. Nuclei stained with Draq5 are shown in blue. Scale bars correspond to 10 μm. Box plots indicate the median, mean, and range of the measured fluorescence signal expressed in arbitrary units (AU) (8–10 image fields with 4–8 cells per image, per sample, n = 3).

To investigate the involvement of PTPRZ1 in mTORC1 activation and protein synthesis, we used *Ptprz1^+/+^* and *Ptprz1^−/−^* LMVEC. Initially, S6K1 (Figure 2A,B) and 4EBP1 (Figure 2A) phosphorylation were found increased in *Ptprz1^−/−^* LMVEC, and rapamycin abolished the enhanced S6K1 phosphorylation in *Ptprz1^−/−^* LMVEC (Figure 2B), suggesting that mTORC1 is activated in the absence of PTPRZ1. Moreover, HIF-1α protein levels are increased in *Ptprz1^−/−^* compared to *Ptprz1^+/+^* LMVEC (Figure S2), further supporting enhanced mTORC1 activity in *Ptprz1^−/−^* LMVEC. Along the same line, protein synthesis is also increased in *Ptprz1^−/−^* compared to *Ptprz1^+/+^* LMVEC (Figure 2C,D), suggesting that PTPRZ1 expression negatively regulates mTORC1 activation and protein synthesis.

### 2.3. PTN Activates mTORC1 and Protein Synthesis in Endothelial Cells through PTPRZ1

**Figure 3:**
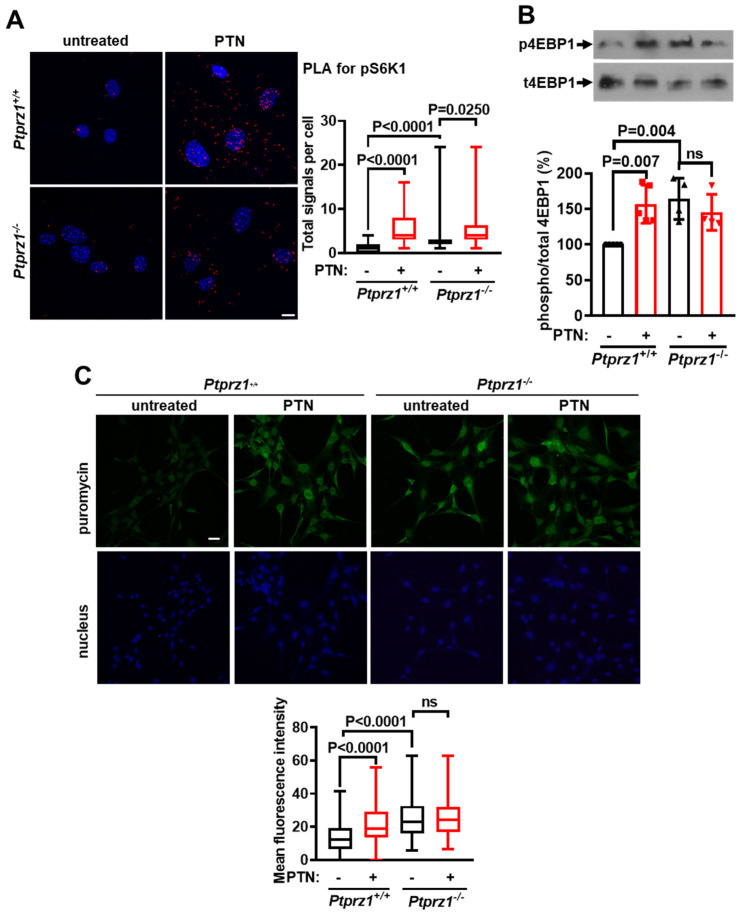
PTN activates mTORC1 and protein synthesis in endothelial cells through PTPRZ1. (**A**) Representative confocal microscopy images and quantification from PLA assay for the detection of the S6K1 phosphorylation sites (red dots), in serum-starved *Ptprz1^+/+^* and *Ptprz1^−/−^* LMVEC stimulated by PTN (100 ng/mL) for 10 min. Nuclei stained with Draq5 are shown in blue. The scale bar corresponds to 10 μm. The box plots indicate the median, mean, and range of detected signals (8–10 image fields with 4–8 cells per image, per sample, n = 3). (**B**) Representative Western blot images of phosphorylated and total 4EBP1 in serum-starved *Ptprz1^+/+^* and *Ptprz1^−/−^* LMVEC stimulated by PTN for 10 min. The bands were quantified, and the results are expressed as the mean ± standard deviation of the % ratio of phosphorylated to total protein compared to the untreated *Ptprz1^+/+^* LMVEC (considered 100%). The bullets on the graph indicate independent assays. (**C**) Representative confocal microscopy images and quantification of the cell-permeable puromycin analog incorporated into newly synthesized proteins of serum-starved *Ptprz1^+/+^* and *Ptprz1^−/−^* LMVEC cultured in the presence or absence of PTN for 2 h. Nuclei stained with Draq5 are shown in blue. The scale bar corresponds to 50 μm. The box plots indicate the median, mean, and range of detected fluorescence signal (8–10 image fields with 4–8 cells per image, per sample, n = 3). ns: non-significant.

**Figure 4:**
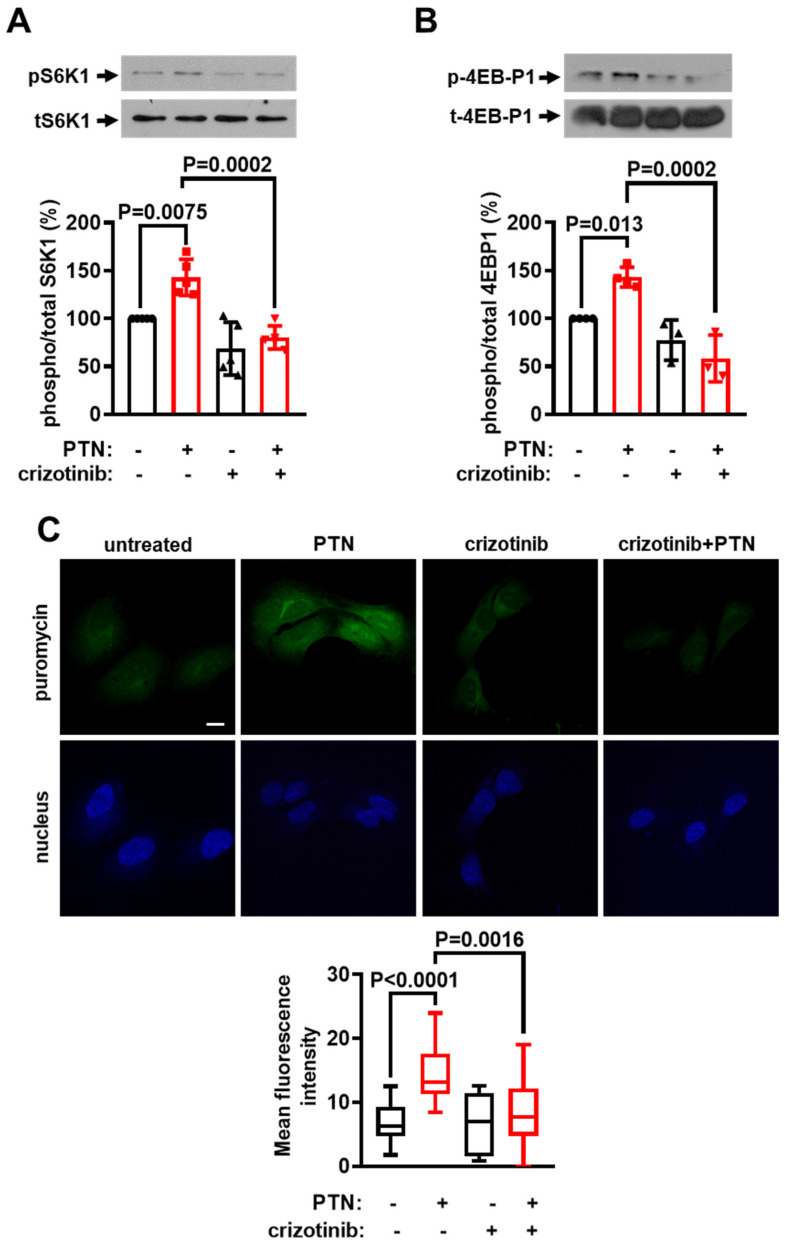
cMet is required for the PTN-induced mTORC1 activation and protein synthesis. Serum-starved HUVEC were stimulated with 100 ng/mL PTN for 10 min in the presence or absence of 1 μM crizotinib. Crizotinib was added to the cells 30 min before PTN stimulation. (**A**,**B**) Representative Western blot images of phosphorylated and total S6K1 and 4EBP1 in HUVEC. The bands were quantified, and the results are presented as the mean ± standard deviation of the % ratio of phosphorylated to total protein compared to the corresponding control (cells incubated with 0.01% DMSO, considered 100%). The bullets on the graphs indicate independent assays. (**C**) Representative confocal microscopy images of serum-starved HUVEC treated with 100 ng/mL PTN in the presence or absence of crizotinib for 2 h. The cell-permeable puromycin analog was added for 30 min to label the newly synthesized peptides (green). The nuclei stained with Draq5 are shown in blue. The scale bar corresponds to 10 μm. Box plots indicate the median, mean, and range of the detected green fluorescence signal per cell (8–10 image fields with 4–8 cells per image, per sample, n = 3).

To investigate whether PTN activates mTORC1 through PTPRZ1, we evaluated the effect of PTN on the phosphorylation levels of S6K1 and 4EBP1 and protein synthesis in *Ptprz1^+/+^* and *Ptprz1 ^−/−^* LMVEC. Like in HUVEC, PTN increased S6K1 (Figure 3A) and 4EBP1 (Figure 3B) phosphorylation and protein synthesis (Figure 3C) in *Ptprz1^+/+^* LMVEC. In unstimulated serum-starved *Ptprz1^−/−^* LMVEC, mTORC1 and protein synthesis were activated and PTN caused no further increase (Figure 3B,C), except in the case of S6K1 phosphorylation (Figure 3A).

### 2.4. cMet Activation Mediates the PTN-Induced mTORC1 Activation Downstream of PTPRZ1

We have recently shown that PTN activates cMet downstream of PTPRZ1 and cMet activation was responsible for PTN-induced endothelial cell migration and the enhanced migration of *Ptprz1^−/−^* compared to *Ptprz1^+/+^* LMVEC [3]. We, therefore, studied whether cMet is also required for PTN-induced mTORC1 activation and protein synthesis in endothelial cells by using the selective cMet inhibitor crizotinib. As shown in Figure 4, crizotinib abolished the PTN-induced phosphorylation of S6K1 (Figure 4A) and 4EBP1 (Figure 4B), and protein synthesis (Figure 4C), suggesting that cMet is upstream of mTORC1 in this signaling pathway. Crizotinib also abolished the enhanced S6K1 phosphorylation observed in *Ptprz1^−/−^* compared to *Ptprz1^+/+^* LMVEC (Figure S3A). The fact that PTN cannot further stimulate the increased mTORC1 activity and protein synthesis in *Ptprz1^−/−^* LMVEC is consistent with the observation that cMet is activated in serum-starved *Ptprz1^−/−^* LMVEC (Figure S3B,C) and that PTN cannot further activate it. PTN significantly activates cMet in *Ptprz1^+/+^* LMVEC (Figure S3C).

### 2.5. mTORC1 Is Required for Endothelial Cell Migration Downstream of the PTN/PTPRZ1 Axis

**Figure 5:**
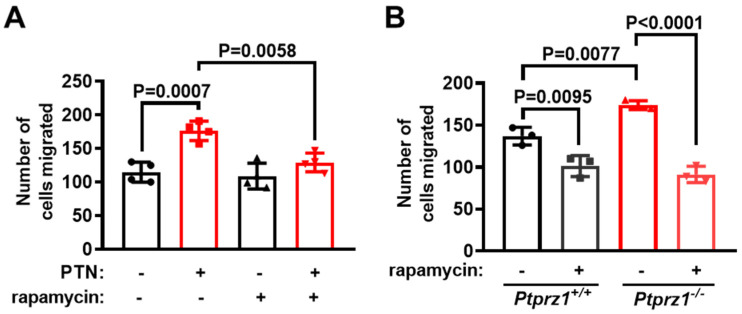
The mTORC1 inhibitor abolishes endothelial cell migration downstream of the PTN/PTPRZ1 axis. (**A**) The number of serum-starved HUVEC that migrated in the presence of PTN (100 ng/mL), rapamycin (20 nM), or their combination. Results are expressed as mean ± standard deviation of the number of cells that migrated through the membrane in the transwell assay. (**B**) *Ptprz1^−/−^* and *Ptprz1^+/+^* LMVEC migration in the presence or absence of rapamycin. Results are expressed as mean ± standard deviation of the number of cells that migrated through the membrane in the transwell assay. In all cases, the bullets on the graphs indicate independent assays and one-way ANOVA was used for statistical analysis.

Το investigate whether the activation of mTORC1 is implicated in endothelial cell migration regulated by PTN and PTPRZ1, we evaluated the impact of rapamycin in endothelial cell migration induced by PTN or PTPRZ1 deletion. Rapamycin abolished the PTN-induced HUVEC migration (Figure 5A), and the increased migration in *Ptprz1^−/−^* compared to *Ptprz1^+/+^* LMVEC (Figure 5B). The effect of rapamycin on migration seems to be due to mTORC1 and not mTORC2 inhibition, since at 4 h, which is the duration of the migration assays, it does not inhibit the phosphorylation of Akt kinase at Ser473 (Figure S4), which is a marker indicative of mTORC2 involvement [14,16].

### 2.6. The Role of α_ν_β_3_ Integrin on mTORC1 Activation

**Figure 6:**
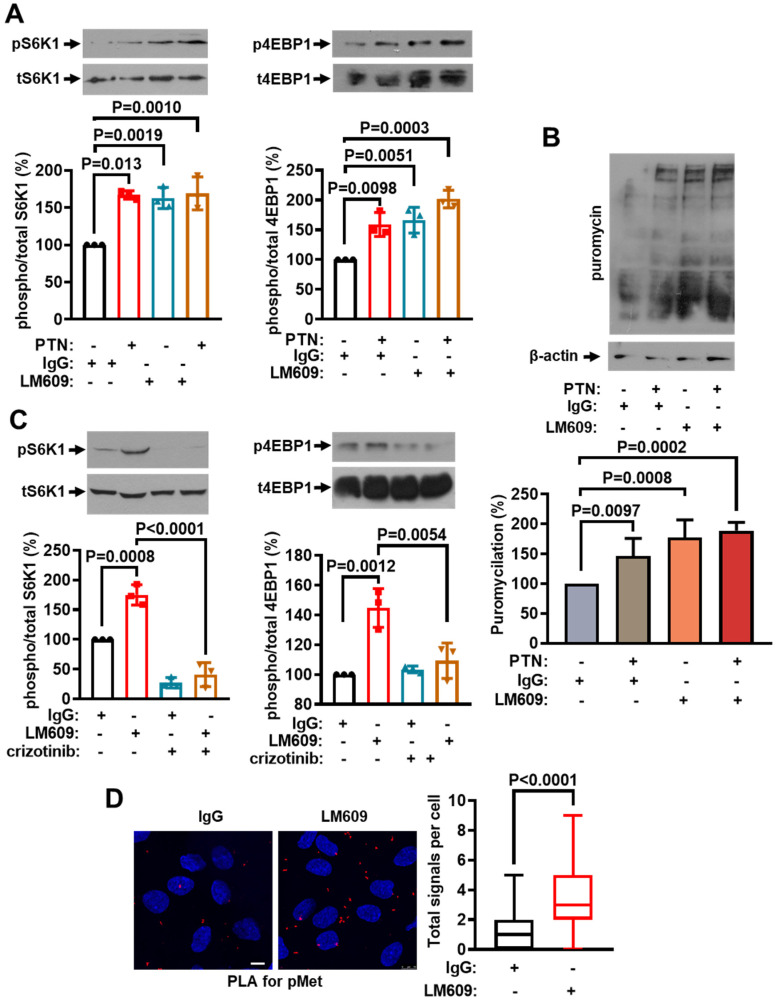
Ligand binding to α_ν_β_3_ integrin activates cMet and mTORC1 in endothelial cells. Serum-starved HUVEC were stimulated with PTN (100 ng/mL), LM609 (10 ng/mL), crizotinib (1 μM) or combinations, for 10 min in A, C, and D and 2 h in B. (**A**,**C**) Representative Western blot images of phosphorylated and total S6K1 and 4EBP1 in HUVEC. The bands were quantified, and the results are presented as the mean ± standard deviation of the % ratio of phosphorylated to total protein compared to the corresponding control (cells incubated with 10 ng/mL IgG, considered 100%). The bullets on the graphs indicate independent assays. (**B**) Representative Western blot images of the newly synthesized peptides labeled with puromycin from total cell lysates from serum-starved HUVEC treated with the tested agents as shown. Puromycin was added for the last 10 min of incubation. Beta (β)-actin was used as a loading control. Results are expressed as the mean ± standard deviation (n = 3) of the % ratio of puromycin incorporation (puromycilation) compared to the control (cells incubated with 10 ng/mL IgG, considered 100%). (**D**) Representative confocal microscopy images and quantification from PLA assay of the tyrosine phosphorylation sites of cMet (red dots) in serum-starved HUVEC treated with IgG or LM609. Nuclei stained with Draq5 are shown in blue. Scale bars correspond to 10 μm. Box plots indicate the median, mean, and range of detected signals (8–10 image fields with 4–8 cells per image, per sample, n = 3).

A critical factor for the PTN-induced endothelial cell migration is the presence of α_ν_β_3_ integrin [4,21,22,23]. The expression levels of α_ν_β_3_ integrin are also important for regulating cMet activation [3]. To investigate the impact of α_ν_β_3_ integrin on the mTORC1 activation in endothelial cells, we used an antibody against α_ν_β_3_ (LM609), which interacts with α_ν_β_3_ and inhibits ligand binding to α_ν_β_3_ [24]. To our surprise, LM609 enhanced S6K and 4EBP1 phosphorylation (Figure 6A) and protein synthesis (Figure 6B) in HUVEC. The effect of LM609 on mTORC1 activation was abolished by crizotinib (Figure 6C), suggesting that cMet activation is upstream of the mTORC1 activation by LM609. Indeed, LM609 significantly enhanced cMet tyrosine phosphorylation (Figure 6D). We also studied the effect of the carboxyterminal PTN peptide, PTN_112−136_, that interacts with α_ν_β_3_ and inhibits PTN binding to α_ν_β_3_ [23]. PTN_112−136_ significantly enhanced cMet tyrosine phosphorylation (Figure S5A) and mTORC1 activity (Figure S5B). All these data suggest that ligand binding to α_ν_β_3_ activates the cMet/mTORC1 pathway that enhances protein synthesis. Combined with our previous data that LM609 and PTN_112−136_ abolish the PTN-induced endothelial cell migration [21,23], they also suggest that although the activation of the cMet/mTORC1 pathway is required, it is not sufficient to enhance endothelial cell migration.

## 3. Discussion

In the present work, we highlighted novel aspects of the PTN/PTPRZ1 signaling axis in endothelial cells and novel functions affected, such as mTORC1 and protein synthesis. Based on our data, PTPRZ1 negatively regulates mTOR activation and mTORC1-dependent protein synthesis in endothelial cells. This is also supported by previous data showing that in fetal human oligodendrocyte progenitor cells, PTPRZ1 deletion or inactivation by PTN enhances their self-renewal competence [25], possibly through the activation of mTOR [18,19]. The activation of mTOR downstream of PTN and PTPRZ1 deletion or inhibition is supported by random observations in more types of cells, although direct evidence is still missing. For example, it has been shown that the suppression of mTOR activity sustains hematopoietic stem cell quiescence [26]. PTPRZ1 genetic deletion or inactivation by PTN enhances the hematopoietic stem cell expansion [27]. PTPRZ1 expression is also increased in human embryonic stem cells [2,28] and is downregulated upon clonal growth and differentiation [28]. mTORC1 activity is low in human embryonic stem cells and enhanced in their differentiated progeny [29]. In the same line, PTPRZ1 is preferentially expressed in glioblastoma stem cells and seems to affect glioblastoma stem cell maintenance, while PTN binding to PTPRZ1 enhances the tumorigenic potential of these cells [30]. Although the role of mTOR in the PTN/PTPRZ1 signaling axis has not been studied in glioblastoma stem cells, it has been shown that mTOR activation contributes to their malignant biological behaviors [31], making it tempting to speculate that mTOR is activated downstream of PTN binding to PTPRZ1 in these cells as well. Finally, we have previously shown that PTPRZ1 expression is decreased in human and murine lung adenocarcinoma [3], in which the mTORC1 signaling pathway is activated [32,33,34,35], further supporting the importance of their potential crosstalk.

Based on the inhibitory effects of rapamycin, our data suggest that mTORC1 and protein synthesis activation are required for PTN-induced endothelial cell migration. Although rapamycin has been shown to also inhibit mTORC2 in cells in vitro [14,15,16], our data support that mTORC1, but not mTORC2, is involved in the described pathway downstream of PTN/PTPRZ1. This is based on the following observations: (a) rapamycin at the concentration used and up to 4 h does not affect the phosphorylation of Akt kinase at Ser473; (b) rapamycin inhibits the phosphorylation of S6K and 4EBP1, which are substrates of mTORC1 [9,10]; (c) rapamycin inhibits protein synthesis which is downstream of mTORC1 [11]. At higher rapamycin concentrations and following prolonged exposure to rapamycin (>24 h), rapamycin may also inhibit mTORC2 and affect endothelial cell viability [15], but this does not seem to be the mechanism involved in our data.

mTOR activity has also been previously linked to endothelial cell migration [36,37], but our data show that it is insufficient to stimulate cell migration. This notion is supported by the data showing that although LM609 and PTN_112–136_ activate mTORC1 (present study), they inhibit endothelial cell migration, whether that is basal or PTN- or VEGFA_165_-stimulated [4,21,23]. Similarly to mTORC1 activation, LM609 and PTN_112–136_ activate cMet (present study), which is also required for PTN- or VEGFA_165_-stimulated endothelial cell migration [3]. However, it seems that cMet activation is not sufficient to stimulate endothelial cell migration. These data highlight the complexity of the signaling pathways that control endothelial cell functions and favor the notion of targeting multiple pathways to inhibit angiogenesis. They also emphasize the need for caution when targeting α_ν_β_3_ to inhibit angiogenesis, providing a potential explanation for the discrepancies in the literature and the failure of such agents in the clinic [38,39,40].

One of the molecules that are affected downstream of mTORC1 is HIF-1α. It has been shown that mTORC1 drives HIF-1α protein accumulation through enhanced transcription of HIF-1α mRNA and/or at the translational level, via 4EBP1 and S6K1 [41]. In *Ptprz1^−/−^* LMVEC, HIF-1α protein levels are increased compared to *Ptprz1^+/+^* LMVEC (this study), although no differences were found at the mRNA level [3], suggesting that HIF-1α is up-regulated in *Ptprz1^−/−^* LMVEC through enhanced mTORC1-dependent protein synthesis. VEGFR2 has also been found upregulated in *Ptprz1^−/−^* LMVEC at the protein but not the mRNA level [3] and mTORC1 activation has been shown to upregulate VEGFR2 at the translational level [42]. RNA sequencing data from *Ptprz1^+/+^* and *Ptprz1^−/−^* LMVEC have uncovered significant differences in only 26 genes [3]. Based on the differences observed in the translation process between these cells, differences in the protein level warrant further study and would be valuable for elucidating the involvement of PTPRZ1 in processes such as ribosome biogenesis, which seems to be regulated mostly through the activation of S6K1 downstream of mTORC1; 4EBP1 phosphorylation has been linked to global protein translation [13,43]. S6K1 phosphorylation is enhanced in *Ptprz1^−/−^* LMVEC but PTN can further activate it, while 4EBP1 phosphorylation and protein synthesis have reached their full activation in the absence of PTN. These data suggest that PTN may fully activate S6K1 through one of its other receptors, e.g., nucleolin, which is well known for its implication in ribosome biogenesis [44]. Nucleolin has been shown to affect mTORC1-dependent protein synthesis, acting at the translational rather than the transcriptional control of several mRNAs implicated in cell metabolism and translation machinery [45]. It has also been shown to mediate PTN-induced mTORC1 activation in fibroblasts [20], and this point will be the focus of future work.

**Figure 7:**
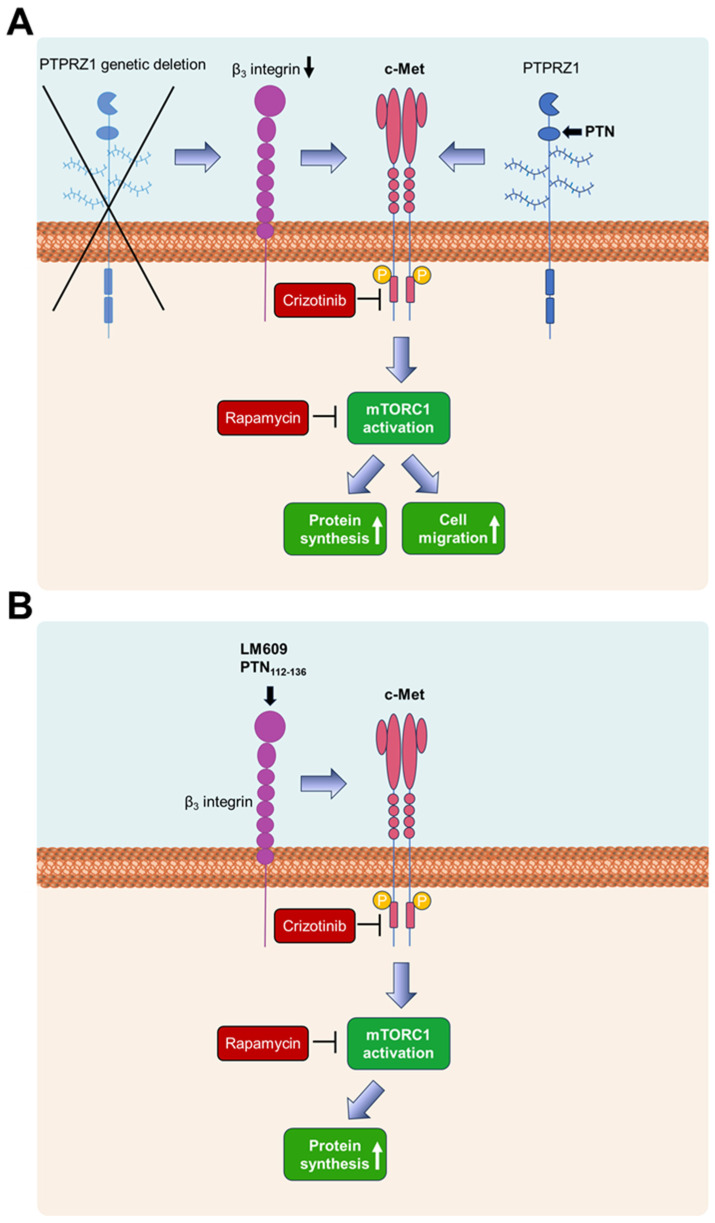
A schematic representation of the PTN/PTPRZ1 signaling pathway that includes α_ν_β_3_ integrin and leads to cMet and mTORC1 activation and enhanced protein synthesis. (**A**) PTN binds to PTPRZ1 and leads to cMet activation, as previously shown [3]. PTPRZ1 deletion is also known to lead to cMet activation [3]. cMet activates mTORC1, which results in enhanced protein synthesis and cell migration. (**B**) LM609 and PTN_112–136_ bind to α_ν_β_3_ integrin and activate cMet, mTORC1, and protein synthesis. The arrows signify up-regulation.

In conclusion, we suggest that PTN binding to PTPRZ1 leads to cMet and mTORC1 activation, enhancing protein synthesis and endothelial cell migration (Figure 7A). PTPRZ1 genetic deletion that results in decreased levels of β_3_ integrin also leads to cMet activation [3], and downstream of cMet, to mTORC1 activation and protein synthesis (Figure 7A). Although it is known that PTN enhances the phosphorylation of β_3_ integrin at Tyr773 in a PTPRZ1-dependent manner [21,22], it is not known whether it affects β_3_ protein levels and, through this, leads to cMet activation. It has been shown that the phosphorylation of β_3_ integrin at Tyr773 may affect β_3_ stability [46] and thus may decrease its levels on the cell membrane, but this needs to be verified by future studies. PTN_112–136_ and LM609 bind to α_ν_β_3_ similarly to PTN [21,23] and activate cMet, mTORC1, and protein synthesis (Figure 7B); however, in contrast to PTN, PTN_112–136_ and LM609 inhibit cell migration [4,23]. These data suggest that although the cMet/mTORC1 pathway is required for endothelial cell migration, it is insufficient to support this function; further studies warrant elucidation of the overall signaling required. Uncovering the signaling molecules involved in regulating PTN/PTPRZ1/α_ν_β_3_-dependent endothelial cell functions will help design more effective and accurate therapeutic approaches for inhibiting angiogenesis, especially considering that VEGFA may activate the same pathway.

## 4. Materials and Methods

### 4.1. Reagents

Crizotinib was from TargetMol (Boston, MA, USA #T1661). Rapamycin was from Santa Cruz Biotechnology, Inc. (Heidelberg, Germany, #sc-3504). All the inhibitors were dissolved in dimethylsulfoxide (DMSO). PTN was from PeproTech, Inc. (Rocky Hill, NJ, USA). Function-blocking monoclonal antibody against α_ν_β_3_ (LM609) was from Merck (#MAB1976, Merck KGaA, Darmstadt, Germany). PTN_112−136_ (LTKPKPQAESKKKKKEGKKQEKMLD) was synthesized as previously described [23].

### 4.2. Cell Culture

HUVEC and LMVEC were isolated and cultured as previously described [3]. The cell culture medium for both was DMEM low glucose containing 15% fetal bovine serum (FBS), 150 g/mL endothelial cell growth supplement (ECGS), 5 units/mL heparin sodium, 100 units/mL penicillin/streptomycin, 100 units/mL amphotericin B, and 2.5 μg/mL gentamycin. Cell cultures were maintained at 37 °C, 5% CO_2,_ and 100% humidity. All experiments were performed with mycoplasma-free cells. Endothelial cells were used in passages 2–4.

### 4.3. Migration Assay

The transwell migration assay was applied using micro chemotaxis chambers from SPL (SPLInsert^TM^ Hanging, #35224, SPL Life Sciences Co., Ltd. 48, Geumgang-ro 2047 beon-gil, Naechon-Myeon, Pocheon-si, Gyeonggi-do, Korea). Endothelial cells were harvested and resuspended in serum-free medium containing 0.25% bovine serum albumin (BSA). An amount of 0.6 mL of medium containing rapamycin 20 nM or PTN (100 ng/mL) or their combination were placed in the lower chamber of each well. In addition, 0.1 mL of the cell suspension containing 10^5^ cells pre-incubated for 15 min with 20 nM rapamycin or with 0.0002% DMSO were loaded on the upper chamber of the transwell. The entire system was incubated for 4 h at 37 °C, 5% CO_2,_ and 100% humidity. The transwell membrane was then incubated in Carson’s phosphate-buffered paraformaldehyde for 20 min at room temperature. The migrated cells were stained with crystal violet (1% solution) and quantified by direct counting in the entire area of each membrane under a light microscope (Optech Microscope Services Ltd., Oxfordshire, UK).

### 4.4. Protein Synthesis Assessment

Protein synthesis was assessed by using the protein Synthesis Assay Kit (Cayman, Ann Arbor, MI, USA, #601100), according to the manufacturer’s instructions. First, 4 × 10^4^ LMVEC or HUVEC were seeded on coverslips in a 24-well plate, cultured in a complete medium for 24 h with confluency up to 80%, and then incubated with starvation medium for 16 h. After treatment of the cells with the tested agents, a cell-permeable puromycin analog containing an alkyne moiety (O-Propargyl-puromycin, OPP) was added to the medium, and cells were incubated for 30 min at 37 °C, 5% CO_2,_ and 100% humidity. Once inside the cells, OPP was incorporated into the C-terminus of translating polypeptide chains, terminating the translation. The truncated C-terminal alkyne-labeled proteins were detected via copper-catalyzed click chemistry. Nuclei were stained with Draq5 (3.3 μM in PBS pH 7.4, Biostatus Limited, Leicestershire, UK; #DRS1000). Cells were mounted with Mowiol 4–88 (Sigma-Aldrich, St. Louis, MO, USA; #81381) and visualized at room temperature with a Leica SP5 (X40 objective) confocal microscope. The mean fluorescence intensity was quantified by using ImageJ. Protein synthesis was also assessed by labeling the newly synthesized peptides with puromycin and by adding puromycin (1 μM final concentration) for 10 min in the culture medium post-treatment, followed by immunoblotting for the puromycilated nascent peptides [47]. The immunoreactive bands were quantified using ImageJ (v1.54h).

### 4.5. Proximity Ligation Assay (PLA)

Either 4 × 10^4^ LMVEC were seeded on coverslips in a 24-well plate or 1.5 × 10^4^ LMVEC were seeded in each well of a 12-well slide (Ibidi GmbH, Gräfelfing, Germany, #81201). PLA was conducted in cells reaching 80% confluency in full medium, without any treatment, or cultured in serum-free medium containing 0.25% BSA for 16 h and then treated with PTN 100 ng/mL for 10 min. All incubations were conducted at 37 °C, 5% CO_2,_ and 100% humidity. Cells were washed once with PBS and fixed with 4% formaldehyde in PBS for 10 min at room temperature. Permeabilization was achieved by 0.05% Triton X-100 in PBS for 5 min at room temperature. In-situ PLAs were performed with the following assay kits from Navinci Diagnostics (Uppsala Science Park, Uppsala, Sweden): (a) NaveniFlex MR using a combination of the rabbit anti-phospho-p70S6 kinase (Thr389) (1:100, Cell Signaling Technology, Danvers, MA, USA; #9234S) and mouse anti-p70S6 kinase (1:100, SantaCruz Biotechnology, Heidelberg, Germany, #sc-8418); (b) Naveni pY Met for detection of tyrosine-phosphorylated cMet, according to the manufacturer’s instructions. Nuclei were stained with Draq5 (as above). Cells were mounted with Mowiol 4–88 and visualized at room temperature with a Leica SP5 (X40 objective) confocal microscope. The quantification was conducted by using ImageJ as previously described [3].

### 4.6. Western Blot Analysis

Cell lysates were analyzed by SDS-PAGE and transferred to PVDF membranes (Porablot PVDF membrane, Macherey-Nagel, Dueren, Germany, #741260), which were incubated in Tris-buffered saline (TBS), pH 7.4, with 0.05% Tween (TBS-T). Blocking was performed by incubating the PVDF membranes in TBS-T containing 5% non-fat dry milk or 3% BSA, for 2 h at room temperature under agitation. The membranes were washed thrice with TBS-T and incubated for 16 h, at 4 °C, under agitation with the 1st antibodies dissolved in TBS-T (containing 5% BSA in the case of the antibodies for the phosphorylated proteins). The membranes were washed 3 times with TBS-T and incubated with HRP-conjugated secondary antibodies (1:2000) in TBS-T for 1 h at room temperature, under agitation. Primary antibodies used were rabbit anti-phospho-4E-BP1 (Ser65), rabbit anti-4E-BP1 (1:1000 and 1:2000, Cell Signaling Technology; #9451T and #9644T), mouse anti-p70S6 kinase (1:1000, SantaCruz Biotechnology, #sc-8418), and rabbit anti-phospho-p70S6 kinase (Thr389) (1:1000, Cell Signaling Technology; #9234S). In the case we studied the phosphorylation of a protein, the membrane was stripped and reprobed to detect the corresponding total protein. For puromycilated nascent peptides detection, an anti-puromycin antibody was used (1:5000 Millipore, Burlington, MA, USA, MABE343, clone 12D10). The membranes were stripped and reprobed for β-actin using a mouse anti-beta actin antibody (1:2000 in TBS-T; Santa Cruz Biotechnology, #sc-58673). The HRP-conjugated secondary antibodies used were anti-mouse IgG (Cell Signaling Technology; #7076) or anti-rabbit IgG (Cell Signaling Technology; #7074). Immunoreactive bands were detected using the SuperSignal West Pico PLUS detection kit (Thermo Fisher Scientific, Waltham, MA, USA, #34577). The immunoreactive bands were quantified using ImageJ.

### 4.7. Statistical Analysis

Data are presented as the mean ± standard deviation (SD) obtained from at least three independent experiments performed in duplicates or triplicates. Statistical analysis was carried out using Student’s unpaired *t*-test to compare values between two groups and one-way ANOVA to compare values among more than two groups. For data sets that did not pass the Kolmogorov–Smirnov normality test, we used the corresponding nonparametric Mann–Whitney (Figure 2B), and Kruskal–Wallis (Figure 1C, Figure 3A,C, Figure 4C, Figure 6D, Figures S3A,C and S5A) tests.

## 5. Conclusions

Our data highlight the involvement of PTN, PTPRZ1, and α_v_β_3_ integrin in mTORC1 activation and protein synthesis in endothelial cells in a cMet-dependent manner. However, although both cMet and mTORC1 are also required for PTN-induced endothelial cell migration, they are insufficient to stimulate endothelial cell migration. More studies are warranted to elucidate how the complex signaling pathways downstream of PTN regulate endothelial cell functions related to angiogenesis and caution is needed when translating data on the use of single-target inhibitors in the clinic.

## Data Availability

All data supporting the findings of this study are available from the corresponding author upon request.

## Supplementary Materials

The following supporting information can be downloaded at: https://www.mdpi.com/article/10.3390/ijms251910839/s1.
