# Supplementary material for: Pleiotrophin Activates cMet- and mTORC1-Dependent Protein Synthesis through PTPRZ1—The Role of ανβ3 Integrin
Source: Int J Mol Sci. 2024 Oct 9;25(19):10839. doi: 10.3390/ijms251910839 (PMC11477150; doi:10.3390/ijms251910839)
Supplement: Supplementary file 1 [file ijms-25-10839-s001.zip › ijms-3201532-supplementary.pdf]

Pleiotrophin Activates cMet and mTORC1-Dependent Protein Synthesis through PTPRZ1—The Role of $\alpha_v\beta_3$ Integrin

Eleni Mourkogianni, Katerina Karavasili, Athanasios Xanthopoulos, Michaela-Karina Enake,

Lydia Menounou, Evangelia Papadimitriou *

Laboratory of Molecular Pharmacology, Department of Pharmacy, University of Patras, 26504 Patras, Greece; eleni9119@yahoo.gr (E.M.); up1079146@upatras.gr (K.K.); athanasiosxan@gmail.com (A.X.); mihaelaenake92@gmail.com (M.-K.E.); up1073880@upnet.gr (L.M.)

Supplementary Methods

Immunofluorescence

$4x10^4$ LMVEC were seeded on coverslips in a 24-well plate and cultured in full medium. After cells reached 80% confluency, they were washed once with PBS and fixed with 4% formaldehyde in PBS for 10 min at room temperature. Permeabilization was achieved by incubating fixed cells in PBS containing 0.1% Triton X-100 for 15 min at room temperature, followed by blocking with PBS containing 3% BSA and 10% FBS for 1 h at room temperature. The cells were then treated with a 1:400 dilution of rabbit anti-HIF-1α antibody (Cell Signaling Technology; #36169), followed by incubation with a 1:500 dilution of Alexa Fluor® 488 chicken anti-rabbit IgG (H+L) (Molecular Probes, Invitrogen, # A-21467). Nuclei were stained with Draq5 (3.3 µM in PBS pH 7.4, Biostatus Limited, Leicestershire, UK; #DRS1000). Cells were mounted with Mowiol 4–88 and visualized at room temperature with a Leica SP5 (x40 objective) confocal microscope.

Western blot

LMVEC lysates were analyzed by SDS-PAGE and transferred to PVDF membranes (Porablot PVDF membrane, Macherey-Nagel, #741260), which were incubated in Tris-buffered saline (TBS), pH 7.4, with 0.05% Tween (TBS-T). Blocking was performed by incubating the PVDF membranes in FastGene Block & Go (NIPPON Genetics EUROPE, Düren, Germany, # FG-CH05) for the phosphorylated cMet, in TBS-T containing 3% BSA for total cMet, or in TBS-T containing 5% non-fat dry milk for all other antibodies, for 2 h at room temperature under agitation. The membranes were washed thrice with TBS-T and incubated for 16 h, at 4°C, under agitation with the 1st antibodies dissolved in TBS-T (containing 5% BSA in the case of the antibodies for the phosphorylated proteins). The membranes were washed 3 times with TBS-T and incubated with HRP-conjugated secondary antibodies (1:2000) in TBS-T for 1 h at room temperature, under agitation. Primary antibodies used were Phospho-Met (Tyr1234/1235) (D26) XP® Rabbit mAb (1:1000, Cell Signaling Technology; #3077), Met (25H2) Mouse mAb (1:1000, Cell Signaling Technology; #3127), anti-phospho-Akt(Ser 473) (1:1000, Cell Signaling Technology; #9271), rabbit anti-Akt (1:1000, Cell Signaling Technology; #9272), rabbit anti-HIF-1α antibody (1:1000, Cell Signaling Technology; #36169) and mouse anti-α-tubulin (1:1,000 in TBS-T, Cell Signaling Technology, #3873). The HRP-conjugated secondary antibodies used were anti-mouse IgG (Cell Signaling Technology; #7076), or anti-rabbit IgG (Cell Signaling Technology; #7074). Immunoreactive bands were detected using the SuperSignal West Pico PLUS detection kit (Thermo Fisher Scientific, #34577). The immunoreactive bands were quantified using ImageJ.

Supplementary Figures

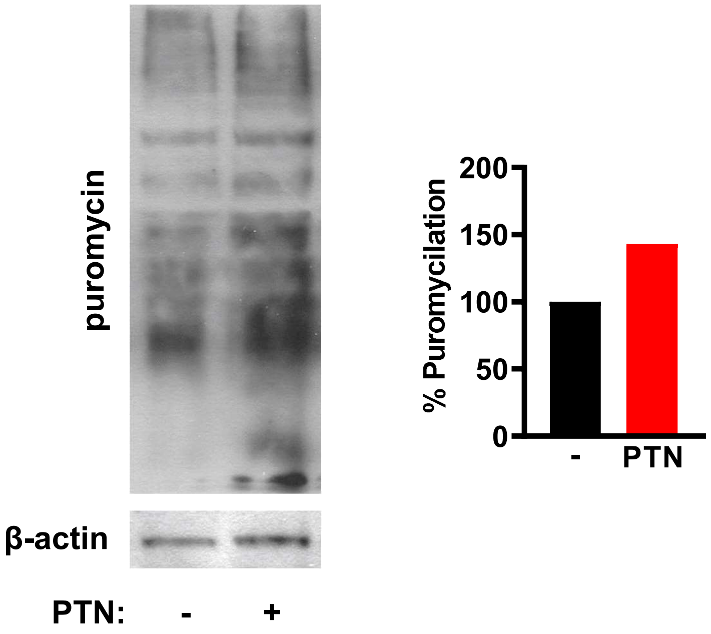

Figure S1. PTN enhances protein synthesis in mouse endothelial cells. Representative Western blot images of the newly synthesized peptides labeled with puromycin from total cell lysates from serum-starved *Ptprz1*$^{+/+}$ LMVEC treated with PTN (100 ng/ml) for 2 h. Puromycin was added for the last 10 min of incubation. Detection was done using an antibody against puromycin. Beta (β)-actin was used as a loading control. Results are expressed as the % ratio of puromycin incorporation (puromycilation) compared to the untreated cells.

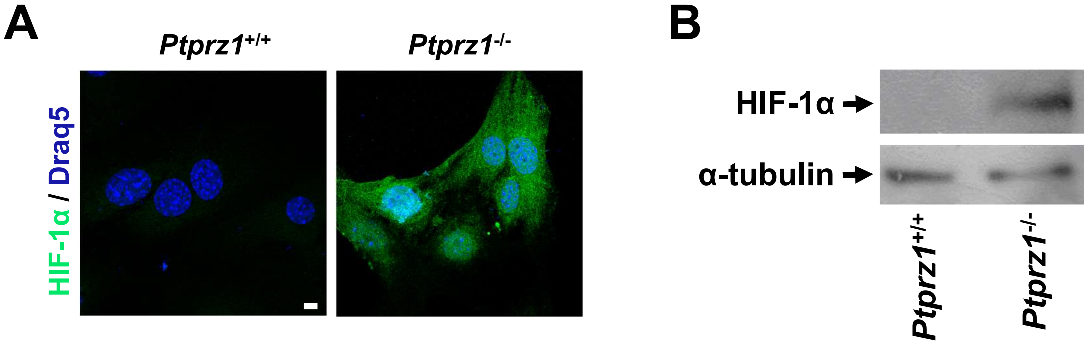

Figure S2. HIF-1α protein levels are increased in *Ptprz1*$^{-/-}$ compared to *Ptprz1*$^{+/+}$ LMVEC. (A) Representative images of immunofluorescence using an antibody selective for HIF1α in *Ptprz1*$^{+/+}$ and *Ptprz1*$^{-/-}$ LMVEC. Cells were fixed and stained with an antibody selective for HIF-1α (green) or Draq5 to stain nuclei (blue). The scale bar corresponds to 10 μm. **(B)** Representative Western blot images of HIF-1α and α-tubulin in *Ptprz1*$^{+/+}$ and *Ptprz1*$^{-/-}$ LMVEC.

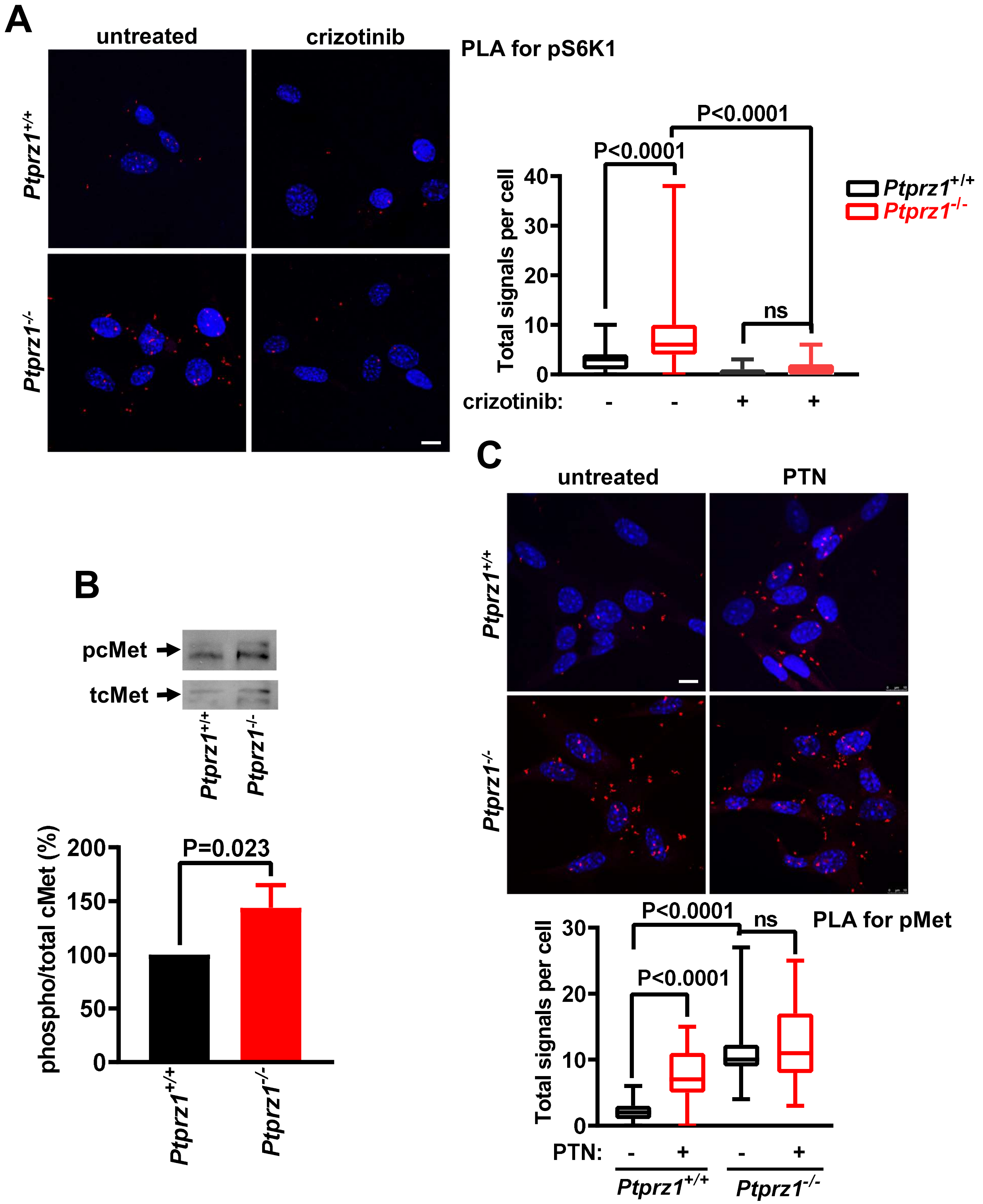

Figure S3. cMet is upstream of mTORC1 in the PTN/PTPRZ1-dependent signaling pathway in LMVEC. (A) Representative confocal microscopy images and quantification from PLA assay for detection of the S6K1 phosphorylation sites (red dots), in *Ptprz1*$^{+/+}$ and *Ptprz1*$^{-/-}$ LMVEC cultured in full medium in the presence or absence of crizotinib (1 μM). Nuclei are shown in blue (Draq5). The scale bar corresponds to 10 μm. The box plots indicate the median, mean, and range of detected signals (8-10 image fields with 4-8 cells per image, per sample, n=3). **(B)** Representative Western blot images of phosphorylated and total cMet in serum-starved *Ptprz1*$^{+/+}$ and *Ptprz1*$^{-/-}$ LMVEC. The bands were quantified, and the results are presented as the mean ± standard deviation (n=3) of the % ratio of phosphorylated to total protein compared to the *Ptprz1*$^{+/+}$ LMVEC (considered 100%). **(C)** Representative confocal microscopy images and quantification from PLA assay of the tyrosine phosphorylation sites of cMet (red dots) in serum-starved *Ptprz1*$^{+/+}$ and *Ptprz1*$^{-/-}$ LMVEC treated with PTN (100 ng/ml). Nuclei are shown in blue (Draq5). Scale bars correspond to 10μm. Box plots indicate the median, mean, and range of detected signals (8-10 image fields with 4-8 cells per image, per sample, n=3).

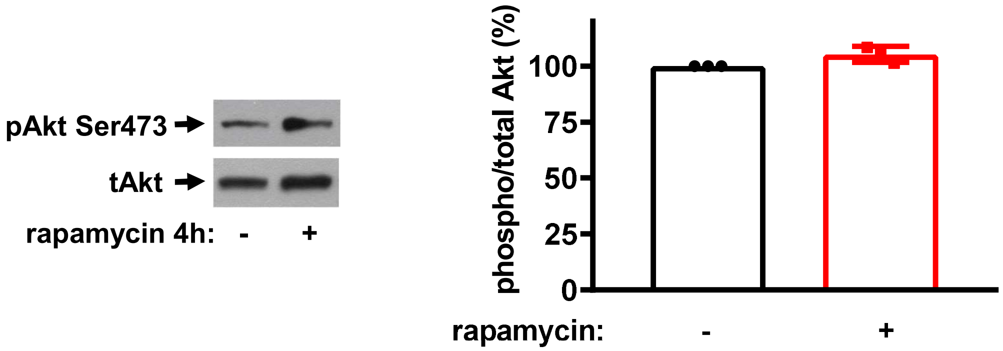

Figure S4. Rapamycin does not affect the phosphorylation of Akt kinase at Ser473. Representative Western blot images of phosphorylated and total Akt kinase in HUVEC incubated with rapamycin 20 nM for 4 h. The bands were quantified, and the results are presented as the mean ± standard deviation of the % ratio of phosphorylated to total protein compared to the corresponding control (considered 100%). The bullets on the graphs indicate independent assays.

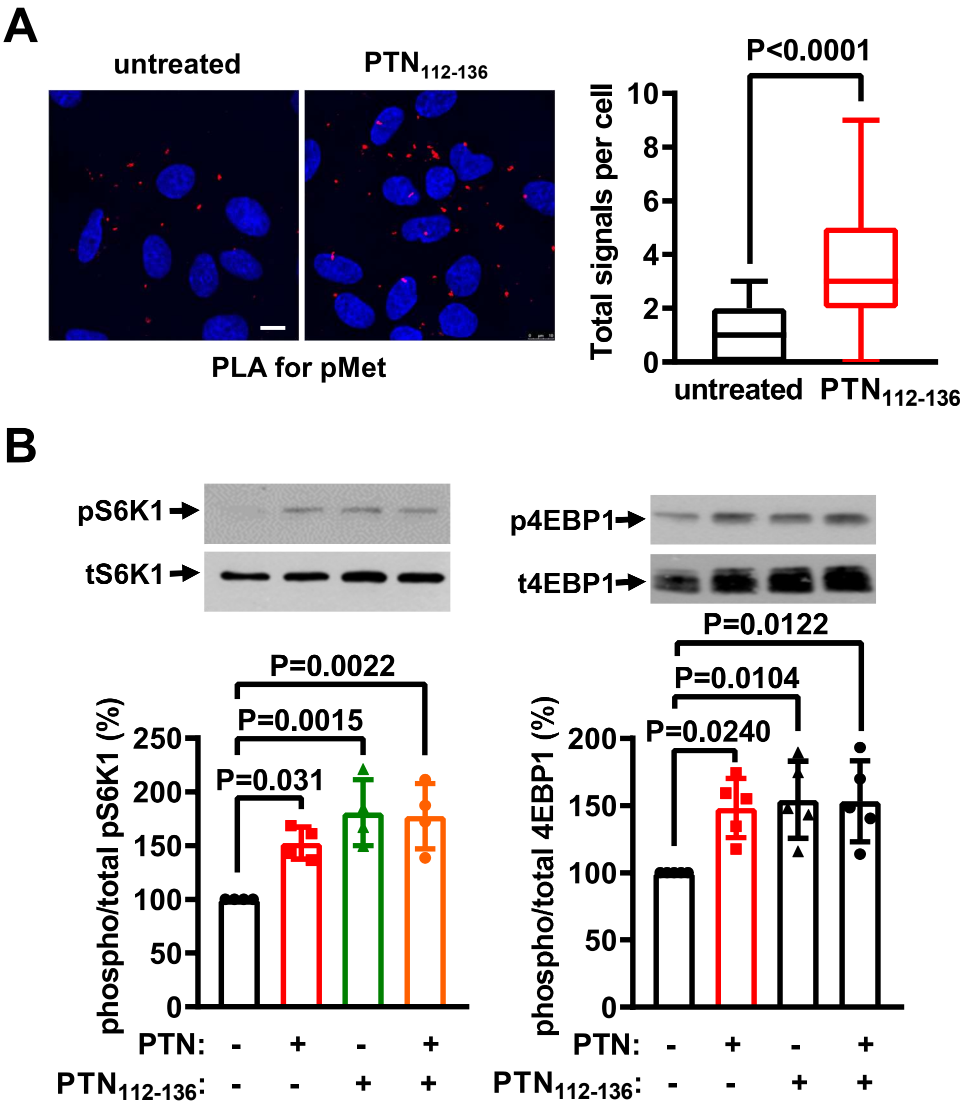

Figure S5. Ligand binding to $\alpha_v\beta_3$ integrin activates cMet and mTORC1 in endothelial cells. Serum-starved HUVEC were stimulated with PTN (100 ng/ml), $PTN_{112-136}$ (100 ng/ml) or their combination for 10 min. **(A)** Representative confocal microscopy images and quantification from PLA assay of the tyrosine phosphorylation sites of cMet (red dots) in serum-starved HUVEC treated with $PTN_{112–136}$. Nuclei stained with Draq5 are shown in blue. Scale bars correspond to 10 μm. Box plots indicate the median, mean, and range of detected signals (8-10 image fields with 4-8 cells per image, per sample, n=3). **(B)** Representative Western blot images of phosphorylated and total S6K1 and 4EBP1 in HUVEC. The bands were quantified, and the results are presented as the mean ± standard deviation of the % ratio of phosphorylated to total protein compared to the corresponding control (considered 100%). The bullets on the graphs indicate independent assays.
